# Patients' Experiences, Needs, and Expectations of Cooperation Between Medical Specialists and Occupational Health Physicians: A Qualitative Study

**DOI:** 10.1097/JOM.0000000000002833

**Published:** 2023-03-07

**Authors:** Ilse J. Oosting, Lana Kluit, Frederieke G. Schaafsma, Annechien Beumer, Coen A. M. van Bennekom, Angela G. E. M. de Boer, Astrid de Wind

**Affiliations:** From the Amsterdam UMC location University of Amsterdam, Public and Occupational Health, Amsterdam, the Netherlands (I.J.O., L.K., F.G.S., A.B., C.A.M.v.B., A.G.E.M.d.B., A.d.W.); Amphia Hospital, Upper Limb Unit Department of Orthopedic Surgery, Breda, the Netherlands (A.B.); Heliomare Rehabilitation Centre, Research and Development, Wijk aan Zee, the Netherlands (C.A.M.v.B.); and Amsterdam Public Health, Societal Participation and Health (L.K., C.A.M.v.B., A.d.W.); and Cancer Center Amsterdam, Cancer Treatment and Quality of Life, Amsterdam, the Netherlands (C.A.M.v.B.).

**Keywords:** clinical medicine, focus groups, occupational medicine, qualitative research, work

## Abstract

Cooperation between clinical and occupational health care practitioners is a key aspect of clinical work-integrating care. This study aimed to gain insight into patients' experiences, needs, and expectations regarding cooperation between medical specialists and occupational health physicians.

**Methods:**

A thematic qualitative study was conducted involving a total of 33 participants in eight online focus groups.

**Results:**

Participants indicated practitioners are currently working in an isolated manner. However, participants desired for partnership between specialists and occupational health physicians to address work-related concerns and showed a need for explanation of the consequences of their diagnosis, so this can be translated into their ability to work.

**Conclusions:**

Currently, cooperation between clinical and occupational health care is lacking. Yet, some participants experienced that these disciplines could complement each other by working together to support patients in work participation.

## Abstract 2

Participants demonstrated the need for a medical professional to address work-related questions, but they did not consent whether a clinical or an occupational healthcare professional was best to consult. Some experienced that these disciplines complemented each other by cooperating to support patients in work participation, but cooperation was generally lacking.

**LEARNING OUTCOMES**

After reading our study, the reader will be able to:
• Describe that patients often experience a lack of cooperation between the medical specialist and the occupational health physician (OHP), which can have a negative effect on a patient's job retention and reintegration opportunities.
• Explain that patients can benefit from the different perspective of an OHP that can enhance clinical health care.
• Recognize that patients under care of a medical specialist need explanation of the impact of their medical diagnosis or diagnostic results on their ability to work and recognize that the OHP is best positioned to provide this explanation to the patient but needs to be supported by a medical specialist with proper information.

The working population in many European countries is aging, and this is accompanied with an increased absolute number of people participating in work with diseases or disorders.^1–3^ When ill, an individual's ability to participate in work may be threatened. Ill health is often associated with a higher probability of labor force exit and reduced work ability,^4,5^ which in turn leads to increased health care use.^6^ It is of societal importance to facilitate work participation for healthy as well as for ill people. From a medical point of view, maintaining employment and a return-to-work plan can promote recovery and rehabilitation and lead to better general health of sick individuals.^7^ Also, employment and return to work can reduce the harmful effects of long-term sickness on the physical and mental health for patients with diseases and can reduce their overall health care consumption.^7,8^ Besides providing income, the ability to work contributes to an individual's quality of life and well-being.^7–10^

Work and health are mutually related. Work can influence health both positively and negatively. For example, adverse work conditions can result in occupational disease, whereas providing a person with support in staying at work can have a positive health impact.^7^ Health can also influence work—especially ill health, which can reduce individuals' ability to work.^4,5^ As such, there is a complex interplay between work and health, which can affect a patient's overall quality of life and well-being.^7^ Because of this complex interplay, employed patients can have questions that relate to both topics.

In clinical work-integrating care (CWIC), there is an understanding that work-related factors can affect health and that medical actions can affect work participation. Health care practitioners pay attention to the interrelationship of work and health, including in medical decision-making, by addressing work in a clinical context. An important aspect of CWIC is cooperation between medical specialists and occupational health physicians (OHPs). Many policy makers have likewise acknowledged that this cooperation is important for improving the effectiveness of prevention, curation, rehabilitation, and reintegration to work and thereby helping to reduce absenteeism and incapacity to work.^11–16^

However, despite the rising attention Towards work participation in clinical health care, cooperation between clinical and occupational health care practitioners is often still lacking.^11,17,18^ There is a need to improve this cooperation to establish CWIC and better serve working patients.^16^ Because the patient is the recipient of this care in the clinical health care setting, it is important to explore patient experiences of this cooperation. It is also important to explore what patients' needs and expectations are. Therefore, the current study aims to gain insight into patients' experiences, needs, and expectations regarding cooperation between clinical and occupational health care practitioners with a focus on medical specialists and OHPs.

## METHODS

### Study Design

We used a thematic analysis according to Braun and Clarke^19^ to explore the patients' needs and expectations regarding this cooperation. COREQ (Consolidated Criteria for Reporting Qualitative Research) was used in this study.^20^ This study was part of a larger qualitative investigation on CWIC. Patients' motives for discussing work with their medical specialists and their needs in CWIC have been extensively described elsewhere.^21^ The data collection for the study took place from September to October 2020. To interpret the study results, it is essential to understand the Dutch occupational health care system—an explanation of which can be found in Box 1.

Box 1. Occupational Health Care in the Netherlands

In the Netherlands, the OHP has two main roles.^17^ First, the OHP acts as gatekeeper to employers during the assessment of employee sick leave, which is regulated by the Gatekeepers Improvement Act.^22^ Second, the OHP provides the employer with expert advice in the field of prevention and health and safety management.^22^ In both roles, the OHP uses his or her expertise to advise the employee and employer on workplace adjustments, work task adjustments, and working hours; refers the employee to other health care practitioners (eg, a medical specialist, psychologist, or physiotherapist); and—during a period of absenteeism—guides the return-to-work process. In practice, most resources are spent on sick leave management, because the Gatekeepers Improvement Act requires that the employer ensure there is an OHP available for the patient, and the patient is obliged to consult this OHP.^22^ Occupational health physicians in the Dutch system most often work for an occupational health service, which have contracts with employers. Some OHPs are self-employed and directly contracted by an employer. This period of contact with the OHP lasts at most 2 years from the start of sick leave, during which the employer pays at least 70% of the patient's salary. Should the patient reach the end of this 2-year period while still not successfully returned to work, the insurance physician—who works for the Employee Insurance Agency commissioned by the Ministry of Social Affairs and Employment—comes into play. The role of the insurance physician is to assess the patients' general work capacity by comparing this with the expected capacity for a person of similar age and educational background, from a social security perspective, in light of a disability benefits application. It should be noted that in the Netherlands not everyone has access to an OHP. Self-employed workers can arrange access to occupational health care themselves, but this is not compulsory by law. Therefore, 80% of self-employed choose not to contract an occupational health service due to, for example, the high costs of these contracts.^23,24^

### Participants and Recruitment

Participants were eligible to enroll in the study if they (1) were of working age (18 to 67 years old); (2) were under the treatment of a medical specialist (ie, physicians with postgraduate specialist training most often working in hospitals such as cardiologists or orthopedic surgeons); (3) were (considered to be) employed or had recently lost employment and were wanting to reenter employment; (4) were able to speak and read Dutch; and (5) had access to the Internet using a personal computer, tablet, or mobile phone with a working camera and microphone. We considered patients eligible regardless whether they had access to an OHP, because we were interested in the experiences, needs, and expectations of patients with as well as without access to an OHP. For example, self-employed patients and job seekers—who in the Dutch system do not have access to an OHP—were eligible. Two sampling methods were used to recruit participants. Convenience sampling was used to recruit participants from outpatient clinics in the departments of cardiology, dermatology, oncology, orthopedics, pulmonology, and rehabilitation in hospitals—general, academic, and specialized clinics—within the network of the researchers. Other participants were purposively recruited based on age, sex, and disease via a patient recruiting platform developed by the Dutch Patient Federation.^25^ This was done to ensure spread among our participants. Recruitment was terminated after data saturation was reached. During the period of data collection, three participants dropped out due to logistical reasons.

### Interview Guide and Data Collection

A multidisciplinary team (L.K., C.A.M.v.B., and A.G.E.M.d.B., all working in the field of occupational health research) created the interview guide based on their expertise. The key questions used during the focus group sessions can be found in Box 2. In total, eight online focus group sessions were conducted using Microsoft Teams,^26^ because the data collection took place during the COVID-19 pandemic. Before each focus group, patients signed a written informed consent form and filled out a brief online questionnaire using Castor EDC^27^ to provide some demographic characteristics (ie, sex, age, educational level, and employment status) and information on treatment-related factors (ie, primary treating medical specialization and years of treatment).

Box 2: Interview Guide (Original Language, Dutch)

1. Have you ever discussed work-related questions with your MS at the hospital?

a. If so, what is your experience with this?

b. If not, what is the reason you never discussed this?

2. What is the most important reason for you to bring up your work during a consultation with your physician in the hospital?

3. Clinical work-integrating care deals with problems at the interface between health and work. What role do you think your MS has in clinical work-integrating care?

4. Have you ever been to an OHP?

a. If not, we continue to question 5b. If so, do you know whether there was contact between your OHP and your medial specialist and what is your experience with this?

5. How do you think cooperation between the hospital and the occupation health physician could improve?

All focus group sessions were moderated by L.K. (female, physician researcher, PhD candidate) and assisted by I.J.O. (female, student of Health Sciences). L.K. was responsible for moving the discussions forward by asking questions and keeping discussions on the topic. I.J.O. took field notes during the sessions and provided technical support. Each focus group started with the moderator repeating the informed consent verbally and setting the ground rules for video conferencing. All focus groups were recorded and transcribed verbatim afterward. After each session, L.K. and I.J.O. filled out a debriefing form to reflect on the session, and participants were asked to fill out an evaluation form as well. The evaluation form contained questions about technical support and gave participants the opportunity to elaborate on responses they had given in the focus group session, although no important additional comments or new information was provided in these forms. Participants received a non-monetary reward for participating in the study.

### Data Analysis

Focus group data were analyzed by conducting a thematic analysis using MAXQDA 2020^28^ and following the guidelines of Braun and Clarke.^19^ Transcription and analysis started directly after each focus group session. Data were analyzed according to a 6-step systematic protocol.^19^ Analysis started with familiarization with the data. In this first step, the researchers (L.K. and I.J.O.) reread the transcripts and noted down their preliminary ideas. Second, initial observations from the transcripts were coded, and the codes with shared concept were grouped according to theme or subtheme. The first four transcripts were independently coded by L.K. and I.J.O. and compared until consensus was reached. The remaining transcripts were coded by L.K. and checked by I.J.O. L.K. and I.J.O. regularly discussed the codes and themes. To support confirmability and reduce bias, one focus group was also coded by an independent researcher. After discussing and eliminating repetitive or irrelevant codes and themes with another independent researcher (A.d.W.), consensus was reached between the researchers on all codes, subthemes, and themes.

## RESULTS

**FIGURE 1:**
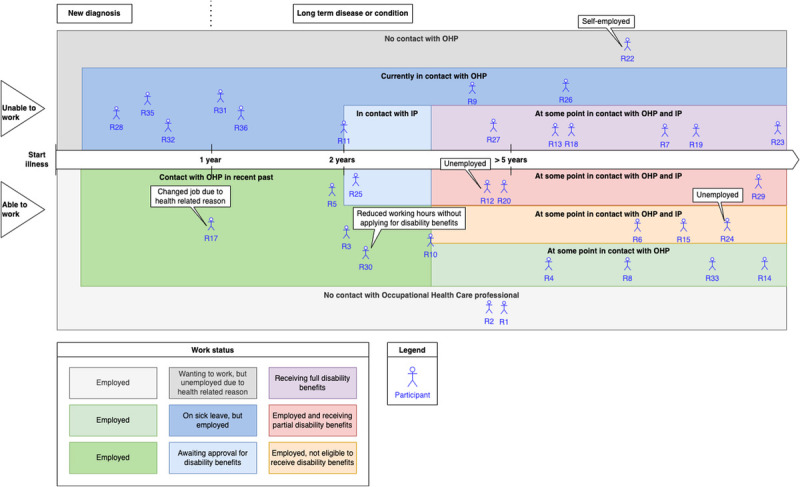
Overview participants in relation to their work status over the course of the patient journey. All patients were under treatment of a medical specialist, at different stages they had been in contact with an occupational health physician (OHP) or insurance physician (IP).

The eight online focus group sessions involved a total of 33 participants. The focus group sessions contained a heterogeneous mix of two to seven patients with different medical histories. The duration of the focus group sessions ranged from 41 to 128 minutes. The sample consisted of 14 men (42%) and 19 women (58%). Ages ranged from 30 to 67 years, with 2 (6%) in the category 30 to 39; 8 (24%) in the category 40 to 49; the majority, 16 (49%), in the category 50 to 59; and 7 (21%) in the category 60 to 67. Figure 1 shows the variability of patients with regard to where they are in the patient journey, from becoming ill to being diagnosed with a (chronic) disease in relation to their current work status. At the time of the focus group session, part of the patients was not able to work because of health circumstances and part of the patients was (partially) able to work. Of the participants, 27 (82%) had experience with one or more OHP(s). In all cases, the contact with the OHP was obliged by the Gatekeepers Improvement Act, because they reached a period of being sick listed for more than 6 weeks. There were no patients in our sample who had been in contact with an OHP for a preventive consultation. Thirteen of them (39% of the total sample) had also been in contact with an insurance physician, because they had been sick listed for a period longer than 2 years. Four participants (12%) had not (yet) been in contact with an OHP. For two participants (6%), it was unclear whether they had spoken to an OHP or an insurance physician when referring to a work-physician—their statements were interpreted with caution.

The results from the focus group data were divided into two major themes: (1) current practices of cooperation between clinical and occupational health care practitioners seem to be that practitioners are working in an isolated manner without interprofessional contact and (2) patients' needs and expectations of cooperation between clinical and occupational health care practitioners: (*a*) patients have a need for support in making the translation of disease to work ability, and (b) patients expect that these practitioners also collaborate to address potential work-related causes for disease. These two themes and the corresponding subthemes are described below, along with illustrative quotes.

### Theme 1: Current Practices of Cooperation Between Clinical and Occupational Health Care Practitioners

#### Practitioners Are Working in an Isolated Manner With Little Interprofessional Contact

Participants experienced that both medical specialists and OHPs are working in their own domains and that there is little interaction between them. The participants had to communicate with each practitioner separately and navigate between them. Although a few participants did mention that their medical specialist had specific advice for the OHP—for example, to perform a work place visit by an occupational therapist—for which they were provided with a letter, most participants were only sent to the OHP by the medical specialist without further ado when asking work-related questions. Most participants did not recall that the medical specialist had any intention for consulting the OHP for the purpose of examining the work-related cause of their disease or make an effort of integrating the OHP in the clinical treatment—for example, to implement (secondary) prevention measures. However, many participants mentioned OHPs needed medical information from the medical specialist for the return-to-work plan, including more information regarding the patient's functional abilities, which could not be deducted from the diagnosis and diagnostics results alone in the experience of some of the participants.

“The physicians, sorry that I say it like that, but they sometimes work quite isolated.” (R30, female, 61, cardiology)

From the participants' experiences, OHPs used three main methods for collecting information from the medical specialist. One method that participants mentioned was OHPs asking the patient to describe the findings of the medical specialist. The second method was to request information transfer from the medical specialist in writing, for which the explicit permission of the patient was required. Alternately, the participant was asked to bring along a medical letter written by the specialist himself or herself, because receiving the requested information from the medical specialist directly from the hospital sometimes took a very long time. In some cases, the patient was asked to put pressure on the specialist to get this information, as illustrated by the following quote:

“So, I had to go after that myself, which was quite annoying, to kind of beg if they could give information to the occupational health service.” (R11, female, 51, oncology)

#### The Complementary Perspectives of Medical Specialists and OHPs

Several participants explained that their OHP had noticed a treatment need for regaining functional abilities to be able to work that their medical specialist had not. Participants suggested that this might have been due to the OHP approaching support for the patient from different perspectives. However, OHPs were not directly involved in the treatment provided by medical specialists. Thus, the participant was asked in these cases to relay the OHP's perspective to the medical specialist by asking for a certain treatment. An example of this was given a participant who asked her pulmonologist for a rehabilitation program in response to her OHP's proposal:

“It was the initiative, the proposal of the OHP, and I myself discussed it with the pulmonologist.” (R6, female, 61, pulmonology)

After this proposal was set in motion by the medical specialist, this participant received the multidisciplinary care she needed. This is exemplary of how such an “interference” can evolve into cooperation between clinical and occupational health care:

“So, for me it was actually a search for ‘how are you going to shape it to be able to keep working?’ And, in the end—mainly due to the rehabilitation, but also in agreement with the pulmonologist and the OHP—they sought out possibilities. That was a complete picture. The medical specialist, the rehabilitation, and the OHP, and work.” (R6, female, 61, pulmonology)

### Theme 2: Patient's Needs and Expectations of Cooperation Between Clinical and Occupational Health Care Practitioners

#### Desire for Partnership Between Health Care Practitioners to Address Work-Related Concerns

It came across throughout all focus groups that work participation was of great importance to the participants. However, because being ill made it hard to maintain employment, participants sought support from a variety of health care practitioners. Participants who received health care that was organized in multidisciplinary teams experienced more attention to work participation, and in particular to work-related concerns, in their treatment from their MS specialist as opposed to those who received treatment from a single medical specialist. However, the OHP was not taking part in these teams. Examples included participants who received health care from a disease-specific multidisciplinary team or participants who were in rehabilitation care. According to these participants, within multidisciplinary teams, medical specialists seemed to use a more personalized approach to treatment, underlining the advantages of cooperation between medical specialists and OHPs, as well as their complementary perspectives. This personalized approach meant that the specialists automatically took into account what the participant's work-related goals were. Working within a multidisciplinary team enabled the medical specialist to refer patients to another health care practitioner within the team who has expertise in providing work-related support. The different health care professionals brought along their own perspective, which resulted in a team treating the patient from a more holistic viewpoint. The participants treated by such multidisciplinary teams reported that each practitioner strengthened and complemented the perspective of the other practitioner in the team, as illustrated by the following quote:

“The multidisciplinary teams […] I have faced that myself in the hospital, where the [disease specific] team operated on me several times. That team consisted, besides several surgeons, also of an occupational therapist, physiotherapist, and rehabilitation doctor, and if such a team cooperates well, which I experienced, this will have a positive contribution. Then you will see there is a one plus one is three thoughts. So, there will be more solutions than coming out of one specialism.” (R13, male, 58, orthopedic surgery)

While work-related concerns could be addressed within these multidisciplinary teams, patients who were not treated by a multidisciplinary team mentioned this as an unmet need. The following participant attested this:

“And then I have been at the [name of the institute] for a while, and there I got a job coach. So, it is indeed what they [the other participants] say, at specialist hospitals of rehabilitation you do get it, but in a normal hospital I get nothing.” (R15, female, 34, endocrinology [pediatric oncology in the past])

With respect to occupational health care, some participants had the experience or expectation that an OHP was not always acting independently in their care. Several participants expressed feeling caught in a triangular relationship between the employer—which they referred to as the “moneylender”—the OHP, and themselves as patient. They mentioned that the Gatekeepers Improvement Act^22^ had caused the “moneylender” to be the one who dictated what happens during the process of care. This resulted in a perception that both the OHP and the patient themselves were subordinate to the employer.

“See, and for me there has been a doubt, is this man here for me, or is he there for my employer? I mean, because he clearly has taken the place of my employer.” (R5, male, 49, orthopedics)

To be able to receive work-related support, many participants wanted a health care practitioner within the clinical health care setting to address their concerns. For some participants, this could be the OHP working within the bounds of the hospital; for others, it could be a nurse or a practitioner already working in the patient's preferred location. Participants suggested that by including an OHP within the multidisciplinary team in clinical care, this physician could then be “independent,” because there would be less involvement from the employer. Others added that if the OHP were part of the multidisciplinary hospital team, medical information could be more easily exchanged between the different disciplines, which would in turn result in less delay in work-related support.

#### Cooperation to Support Patients in Translating Disease Diagnosis Into Work Ability

Our study participants expressed a general need for better explanations of the implications of their medical diagnoses and diagnostic results on their ability to work. They noted that the OHP functions as the medical expert best positioned to do so, because the medical specialist does not have enough knowledge of various types of jobs. Therefore, the participants expected OHPs to be capable of translating information about a patient's disease and treatment into an understanding of the limitations these may present to the patient's ability to function at work. Participants believed this clarity around their ability to work would lead to more effective return to work and sustainable employability.

“It would be nice if he can make the translation into work, but I think that can't be the medical specialist in all cases. He can speak the same language as, for instance, the OHP, who also knows the medical language, but can also make the translation into work capacity, which is needed to do your job, and keep doing your job.” (R8, female, 43, gastroenterology)

However, in the experience of some participants, the OHP sometimes lacked knowledge of their specific disease or treatment. These participants did not feel supported by their OHP. They explained that it would help if the OHP gathered information from the medical specialist, facilitating an exchange of expertise.

“And I would have thought that I would get that from the occupational physician. That he would find out, together with me and with the information from all the specialists, how we can figure that out. Unfortunately, my occupational physician did not do that.”

R19, female, 44, pulmonology

## DISCUSSION

The aim of this study was to gain insight into patients' experiences, needs, and expectations regarding cooperation between clinical and occupational health care practitioners with a focus on the medical specialist and OHP. Our participants experienced receiving separate care from their medical specialists and OHPs, which is in line with the separation between the clinical and occupational health care system in the Netherlands as it is currently established.^17^ All participants expressed the need for access to a practitioner who can address their work-related questions, but they debated who this person could be and where this person would be best placed. Some participants believed that OHPs should be included in a multidisciplinary team within the clinical health care setting. Others reported having found another health care provider to support them with their work-related needs. Overall, our participants expressed a desire for greater collaboration between different health care practitioners—including cooperation between clinical and occupational health care practitioners—to support them with their work-related concerns.

As was confirmed by our participants, it is important that the medical specialist and OHP work together, providing that they can complement each other's expertise. This entails two elements: first, by treating the patient from a biopsychosocial approach, including a clinical and an occupational health perspective, work-related concerns will be addressed in a more adequate way,^29^ and consequently, the patient will be better served. Second, complementing each other's expertise furthermore enables the medical specialist and OHP to support patients in translating what their diagnosis and disease mean for their work ability.

In the current practice, the medical specialist and OHP most often do not succeed in complementing each other's expertise. As participants mentioned, one component for sustaining this could be that some patients are doubtful about the independence of the OHP and fear negative consequences for continuation of their employment when certain information would reach the OHP.^30–33^ This patient's doubt could have repercussions on the medical specialists actions by influencing their opinion as well.^32,34^ This perceived lack of independence could be related to the way in which the Dutch health care system is set up and functions. All citizens in the Netherlands have access to clinical health care, which is financed by mandatory health insurance for each individual, whereas occupational health care is organized differently. The Dutch system has provided an incentive to reduce unjustified sickness benefits in the past years through the implementation of the Gatekeepers Improvement Act,^22^ through which employers are incentivized to support their employees in returning to and staying at work—as explained in Box 1. On top of that, employers could benefit from early return to work, because this could eliminate the cost of hiring and training a replacement.^35,36^ However, this change has caused some patients to experience a feeling of pressure from their employer, which they feel the OHP may reinforce.

The lack of cooperation between the medical specialist and OHP also presents challenges to patients who are self-employed or unemployed. These patients may receive limited or even no support from an OHP because there is no employer involved, which makes the Gatekeepers Improvement Act not apply.^32^ This results in the fact that they often cannot access occupational health care, because they are often not insured for this care otherwise.^17,24^ Although some of these patients received support regarding their work-related concerns within the clinical health care setting—particularly in care provided by multidisciplinary teams or in rehabilitation—most of these patients fell through the gaps of the Dutch health care system, because they were not able to receive the care and support they actually needed.

One could argue that a change in the organization of payment systems for clinical and occupational health care may result in an improved cooperation and exchange of information between clinical and occupational health care.^37^ Structural changes in a health care system are difficult to implement and would probably not be achievable in the future. To support patients in translating their disease diagnosis into an understanding of their work ability without changing current policies, we suggest optimizing communication between clinical and occupational health care. The World Health Organization's International Classification of Functioning (ICF) model might be used as a conceptual framework for stimulating cooperation and bridging the gap between medical specialists and OHPs.^38^ Thinking in terms of the ICF model could support communication between the OHP and medical specialist by providing a framework to enable them to communicate about the treatment from a biopsychosocial approach, integrating the clinical and an occupational health care perspective.^39^ This could help the medical specialist to serve in the patients' needs toward work-related questions and actively reach out to the OHP and vice versa.

### Methodological Considerations

A strength of this study was the heterogeneous group of participants with varying medical histories and experiences with clinical practice. The combination of convenience and purposive sampling resulted in two groups of participants that were slightly diverse from each other. By combining the two strategies, we were able to recruit participants from different patient populations. Overall, the group of patients forming the convenience sample strategy consisted of more patients with newly diagnosed disease, and the purposive sample consisted of more patients with chronic disease. However, patients with newly diagnosed disease and those with chronic disease were recruited via both strategies and therefore in the end equally balanced in our total sample.

During the analysis, we took into account that many participants from the more chronically ill patient group were already more experienced in health care and were often depending on (partial) disability benefits. When depending on disability benefits, one has undergone a work disability assessment performed by an insurance physician (see Box 1). For two participants, the difference between an insurance physician and an OHP was not self-evident, which may have biased their opinions regarding occupational health care. In addition, because of being a patient with chronic disease, one might become more reflective—and sometimes critical—of the health care system, which could have also have introduced some form of bias. For example, having a disability pension suggests a nonsuccessful return-to-work strategy in the past, which may have been reflected in their opinions. Furthermore, four participants had not (yet) been in contact with an OHP but were involved in the focus group discussions. However, this was taken into account during the analysis by discriminating between patients' actual experiences and their perceptions only. Despite the differences between experienced and newly diagnosed patients, we noticed that most of participants faced the same work-related concerns and therefore had similar experiences or perceptions.

Because of the COVID-19 pandemic, we were restricted to conducting our focus group sessions in an online environment. This provided a physical barrier at the time because social distancing measures were in place, and it therefore created safety for our participants. Additional benefits were that it allowed us to involve participants located anywhere in the country, it was less expensive and more time efficient, and participants may have felt more at ease to make comments because they were not in front of real people.^40^ The use of Microsoft Teams allowed us at the time of recording to face a maximum of only nine people without hiding anyone. Therefore, we chose to invite fewer participants per focus group than a normal size of 8 to 12 persons, because we found it important to be able to face everyone including the moderator and observer during the session. We experienced more group dynamics and more cohesion between participants in the smaller groups of four to five persons compared with one focus group containing a maximum number of seven participants.

A limitation to the study design was the short time frame in which the data collection was performed. Because of conducting all focus groups within 2 months, there was little time for reflection and analysis in between sessions. As a consequence, the first overview of themes and subthemes was deducted after conducting the last focus group. However, no new theoretical insights emerged while analyzing the last two focus groups, so we were able to conclude that data saturation was reached in this study.^41^ This study was part of a larger qualitative investigation on CWIC, in which patients' motives for discussing work with their medical specialists and their needs in CWIC were investigated. As a result, this study was limited to the patients' perspectives from the viewpoint of clinical health care.

Finally, it should be noted that our study was conducted within the Dutch heath care system, which has some specific characteristics that are not necessarily applicable to health care systems in other countries. One specific element is the strict separation in payment systems between occupational and clinical health care. Because of this separation, OHPs cannot be contracted within a general hospital because they receive outside financial support. This might affect the transferability of our results to other countries. Nevertheless, patients experienced the freedom to discuss work with multidisciplinary teams regardless of the involvement of an OHP due to the multiple perspectives the health care professionals brought. This finding could be applicable to other countries as well.

### Implications for Further Research and Practice

In the coming years, the numbers of older people of working age in society will increase,^1–3^ which means that the amount of people working with a (chronic) disease will likewise increase. It is of great importance to support work sustainability in the future and to support patients at work. Currently, cooperation between clinical and occupational health care is lacking. As a consequence, job retention and reintegration opportunities are being missed in current practice. Therefore, cooperation within CWIC should be enhanced. Our participants expressed the desire to have someone to whom they can address their work-related concerns. Occupational health physicians already possess the expertise to help these patients, but apparently are now positioned in the Dutch health care system in such a way that not all patients perceive their advice and judgments as independent and supportive. Some participants therefore expressed the desire that OHPs be integrated directly into the clinical system and the hospital itself. This way, the medical specialist and OHP would be literally and figuratively closer to each other, and it would be easier for them to cooperate, which could particularly serve patients with complex medical and work-related problems. However, most patients do not have complex problems; thus, another solution could be to improve the image patients have of OHPs. This might enhance the usage of occupational health care within the current system and also improve communication between medical specialist and OHP because the patient might then ask for it instead of inhibiting it. Improving the image of OHPs could be a topic of further research.

Although placing the OHP within the clinical health care system seems to be a quite straightforward solution, it would require a change in the broader Dutch health care system, which is not easy to accomplish. Meanwhile, another practical solution could be tried first. As mentioned previously, the ICF model and classification could be implemented to provide a framework and language to improve information exchange between the medical specialist and OHP, which could in turn support patients in translating their diseases into the implications of their functioning at work. Having a more structured way of sharing information about patients' functional abilities could provide more clarity with regard to the expectations of the medical specialist. Making and implementing this framework in clinical practice could be a topic of future research.

## CONCLUSION

All participants showed the need to have access to a health care practitioner to address their work-related questions, but they debated who this person should be and where this person would best be situated. Even though most participants experienced a lack of cooperation between the medical specialist and OHP, they also anticipated how these disciplines could complement each other to support patients' work participation. Within the context of Dutch policies aiming to support (chronically ill) people to remain at work as much as possible, patients sometimes experience or expect pressure from an OHP. This pressure causes friction in the perceived independence of the OHP by patients. Yet, patients also perceive the OHP as well suited to provide occupational health care. Therefore, it may be useful to educate patients in general about the layout of the Dutch health care system and the different roles of the medical specialist and OHP herein. Specifically, the self-employed should be informed about the possibilities to insure for occupational health. Finally, to enhance cooperation between clinical and occupational health care as a key aspect of CWIC, the medical specialist and the OHP would both need to communicate with the same language. This will benefit sustainable work participation for patients.
